# Elementary, my dear Cameron

**DOI:** 10.1186/2041-2223-2-5

**Published:** 2011-02-01

**Authors:** Mark A Jobling

**Affiliations:** 1 Department of Genetics, University of Leicester, Leicester, UK

## 

All science is about creativity and ingenuity: coming up with a good way to answer a tricky question. But with forensic science, somehow these qualities take on an additional lustre. In part, this is the romance of the whodunit - tales of Dupin, Holmes or Poirot; in part, it is the application of so many diverse scientific disciplines to the same ultimate goal; and, in part, it is the knowledge that the effort can bring about a public good, in the shape of justice.

I read two papers recently that reminded me of this ingenuity. The first describes the application of a highly abstract and academic discipline, molecular phylogenetics, to support two convictions for assault [1] and the second the application of a spin-off from immunology to determine the age of a person from a sample of their blood alone [2].

The weapon in the assault cases [1] was, despite its small size, a deadly one - the virus HIV-1. The question being asked was about the direction of transmission of the virus among a set of infected individuals: in particular, does the phylogenetic evidence support the hypothesis that the defendant (who knew himself to be HIV-positive in each case) was responsible for the infections carried by a number of his sexual partners? Two aspects of HIV-1 biology were exploited in order to address this: first, the virus has a high mutation rate (about 3 × 10^-5 ^per base per replication cycle) and so, during proliferation within an infected person, a diverse set of HIV-1 sequences arises; and second, when one person infects another, the population of viruses goes through a strong genetic bottleneck, so that three-quarters of new infections are each established by a single virus.

In each case the sample identity was blinded and parts of the HIV-1 genome sequenced in the putative offender and victims. A phylogenetic tree showed that sequences from one DNA donor contained subsets that were more closely related to the sequences carried by other donors than they were to other subsets in the same donor. This property ('paraphyly') demonstrates that one DNA donor is the source of the infections in the others. In each case the inferred source was revealed to be the defendant and the DNA evidence contributed to the conviction.

The second paper [2] exploits the curious fact that, as a person ages, their thymus shrinks. This organ is the home of T-lymphocyte development and, as these cells mature, they rearrange the genes encoding T-cell receptors (TCRs), ejecting a DNA circle in the process. The concentration of one specific circle can be measured in a blood sample and normalized by comparing it with a control gene sequence. This normalized concentration can be compared with the age of the blood donor, with the expectation that the older the donor, the smaller their thymus and the less of the DNA circle their blood should contain. The correlation is not perfect, but nonetheless impressive, allowing >89% accuracy in assigning a donor to a particular 20-year window of age. The method also works on blood that is old or in small amounts and so provides another tool in the forensic investigative toolbox when no suspect is available.

Fostering this kind of creativity and ingenuity in forensic science requires investment. Of course, research in universities funded through diverse sources is important here - indeed, both the examples given above come from university-based groups. However, state-funded forensic science services also play a major role in the development of novel methods, as well as in setting standards. This is something the UK government appears not to appreciate because it has recently announced plans to close its Forensic Science Service (FSS). The FSS traces its origins to the 1930 s and was a key player in the development of DNA profiling and the establishment of the first national DNA database, begun in 1995. Over the last 10 years the FSS has undergone stepwise changes of status that, in effect, have transformed it into a company that must compete with other suppliers of forensic services that do not have its responsibilities, research capability or scope. Surprise, surprise, it has not competed effectively in the marketplace and stands accused today of losing too much money.

Successive governments have behaved with cynicism here - acting, as Oscar Wilde put it, as if knowing the price of everything but the value of nothing. A market based on the cheapest possible provision of standard forensic services will fail the UK's criminal justice system when specialized and innovative methods are needed for particular cases. David Cameron's coalition has already been forced into some embarrassing U-turns, including a change of heart on the axing of highly successful schools sports programmes in the year before the London Olympic Games. Pressure for them to reconsider their position on the FSS has been increasing from the international community of forensic scientists [3] and in the form of a growing public petition demanding a debate in parliament. Concerned readers should add their voices to this chorus: Cameron is making a mistake.

The Editors-in-Chief of the journal have also commented on the closure of the FSS in an editorial, available here http://www.investigativegenetics.com/content/2/1/4.
